# Epithelial-mesenchymal transition of cancer cells using bioengineered hybrid scaffold composed of hydrogel/3D-fibrous framework

**DOI:** 10.1038/s41598-019-45384-9

**Published:** 2019-06-20

**Authors:** Mintu Pal, Huizhi Chen, Bae Hoon Lee, Justin Yin Hao Lee, Yun Sheng Yip, Nguan Soon Tan, Lay Poh Tan

**Affiliations:** 1 School of Materials Science and Engineering, Nanyang Technological University, 50 Nanyang Avenue, Singapore, 639798 Singapore; 2 Biological Sciences and Technology Division, Biotechnology Group, CSIR-North East Institute of Science & Technology, Academy of Scientific and Innovative Research, Jorhat, Assam 785006 India; 3 School of Biomedical Engineering, School of Ophthalmology and Optometry, Eye Hospital, Wenzhou Medical University, Wenzhou, 325027 China; 4 Lee Kong Chian School of Medicine, Nanyang Technological University, 11 Mandalay Road, Singapore, 308232 Singapore; 5 School of Biological Sciences, Nanyang Technological University, 60 Nanyang Drive, Singapore, 637551 Singapore

**Keywords:** Biomaterials - cells, Cancer models

## Abstract

Cancer cells undergoing epithelial-mesenchymal transition (EMT) acquire stem cell-like phenotype associated with malignant behaviour, chemoresistance, and relapse. Current two-dimensional (2D) *in-vitro* culture models of tumorigenesis are inadequate to replicate the complexity of *in-vivo* microenvironment. Therefore, the generation of functional three-dimensional (3D) constructs is a fundamental prerequisite to form multi-cellular tumour spheroids for studying basic pathological mechanisms. In this study, we focused on two major points (i) designing and fabrication of 3D hybrid scaffolds comprising electrospun fibers with cancer cells embedded within hydrogels, and (ii) determining the potential roles of 3D hybrid scaffolds associated with EMT in cancer progression and metastasis. Our findings revealed that 3D hybrid scaffold enhances cell proliferation and induces cancer cells to undergo EMT, as demonstrated by significant up-regulation of EMT associated transcriptional factors including Snail1, Zeb1, and Twist2; and mesenchymal markers whereas epithelial marker, E-Cadherin was downregulated. Remarkably, this induction is independent of cancer cell-type as similar results were obtained for breast cancer cells, MDA-MB-231 and gastric cancer cells, MKN74. Moreover, the hybrid scaffolds enrich aggressive cancer cells with stem cell properties. We showed that our 3D scaffolds could trigger EMT of cancer cells which could provide a useful model for studying anticancer therapeutics against metastasis.

## Introduction

Cancer metastasis is the hallmark of tumour malignancy that begins with the process of epithelial-mesenchymal transition (EMT), where well polarized epithelial cells are converted into non-polarized mesenchymal cells acquiring invasion and motility properties. Importantly, 90% of cancer-related death is attributed to metastasis. Various environmental factors (e.g., hypoxia), growth factors (e.g., TGF-β1) and cell-extracellular matrix (ECM) can trigger EMT. Epithelial cancer cells undergoing EMT adopt a cancer stem cell (CSC)-like phenotype and is uniquely capable of seeding new tumours^1–3^. The identification of the EMT program as a critical regulator of the CSC phenotype offers an opportunity to investigate the nature of intratumoral heterogeneity and a possible mechanistic basis for anticancer drug resistance^4,5^. Gathering evidence indicates that conventional chemotherapies often fail to eradicate carcinoma cells that have entered the CSC state via activation of the EMT program, thereby permitting CSC-mediated clinical relapse^1^. Thus far, our current understanding of the link between the EMT program and the CSC state has been derived largely from studies performed on two-dimensional (2D) cell culture studies, which poorly represent the complexity of *in-vivo* tumour due to the lack of appropriate cell-ECM and cell-cell interactions. Furthermore, current research, mainly 2D, is unable to isolate and enrich CSCs population in *in-vitro* conditions effectively^6,7^. Thus, there has been extensive research on developing three-dimension (3D) cell culture models using scaffolds and scaffold-free techniques that better mimicked the *in-vivo* tumour microenvironment which facilitates neoplastic growth and metastasis^8–10^. Indeed, Gkretsi *et al*. and Cox *et al*. highlighted the importance of remodeling components of the tumor microenvironment to enhance cancer therapy^11,12^. Recently, Stylianopoulos *et al*. emphasized the reengineering of the physical microenvironment of the tumor to improve drug delivery and efficacy^13^. These 3D models aim to provide a salubrious microenvironment for the EMT process and maintenance of CSCs, which would be essential for studying the biological response to various types of therapeutic drugs to inhibit the growth of CSCs.

Great progress in the field of biomaterial and scaffold engineering in the past decades spark the creation and development of organized tissue constructs^14–17^. Currently, there are several processing techniques to design and fabricate 3D scaffold as tissue engineering implants, such as solvent casting/particulate leaching technique^18^, emulsion templating^19^, gas foaming technology^20^ and liquid-collecting electrospinning^21^. The porous scaffolds are able to provide a large internal volume and 3D space where cells can aggregate and form tissue-like structures. In particular, electrospinning is a versatile polymer processing method commonly used to fabricate micro/nanofibers structures. These fibers form a non-woven fabric that highly mimics the fibrous network of native ECM with fiber diameters that is close to ECM fibrils (i.e., from nanometer to micrometer)^22^. Electrospun fiber matrices have been shown to offer morphologic cues that result in enhanced cell responses^23,24^. These fibrous scaffolds have presented improved cell attachment, proliferation, migration, and gene expression signature^25–27^. However, the conventional electrospinning usually produces 2D dense mats rather than 3D porous structures, which impedes cell infiltration into the scaffold. The liquid-collector method overcomes this shortcoming and allows the fabrication of 3D fibrous scaffolds with high porosity via electrospinning^21^. Apart from scaffold architecture, the selection of appropriate biomaterials is equally important. Typically, the native ECM is comprised of proteins, proteoglycans, and other soluble molecules. ECM provides mainly the structural support and regulatory milieu for cellular growth and communications with other cells. Synthetic polyesters such as Poly Lactic-co-Glycolic Acid (PLGA) are biodegradable, biocompatible and have been approved by the Food and Drug Administration. However synthetic materials usually lack adhesion motifs that promote favorable cell-material interactions.

Therefore, in this study, we utilized photopolymerizable gelatin methacrylamide (GelMA) hydrogel to provide a conducive biochemical environment and PLGA fibrous scaffold to confer filamentous nature of natural ECM. Together with GelMA hydrogel, our biomimetic 3D hybrid scaffold potentiates EMT of a subpopulation of cancer cells and supports the growth of other subpopulations cancer cells. This heterogeneity highlights the strength of our 3D scaffold to realistically mimic the *in-vivo* situation, where tumors are heterogeneous subpopulations of cells. This scaffold recapitulated tumour microenvironments conducive for the metastasis process of a polarized gastric cancer cell line, as well as enriched and maintained CSC-like characteristic of highly invasive triple-negative breast cancer cells^28,29^. As 90% of cancer-related death is attributed to metastasis, our model is useful for the study of anticancer therapeutics against metastasis that accounts for therapy resistance. Our findings could also provide a platform for scientists to study mechanosignalling in tumor progression in 3D.

## Results

### Materials and scaffold characterization

As shown in Supplementary Fig. S1A,B, the PLGA 3D fibrous scaffold is highly porous with fiber diameters ranging from 1.0 to 1.8 µm and an average fiber diameter of 1.6 ± 0.13 µm. The pore sizes ranged from 5 to 40 µm, where most of them were between 5 and 20 µm with an average pore size of 14.54 ± 6.47 µm (Supplementary Fig. S1C). As PLGA has a relatively hydrophobic nature^30,31^, GelMA was added to the scaffold to provide cell-adhesive ligands for cell recognition and promote better cell infiltration. Synthesized GelMA was characterized by NMR as shown in Supplementary Fig. S2. Comparing the spectrum of GelMA with unmodified gelatin, new functional groups formed in GelMA were marked as orange “a” and green “c” in Supplementary Fig. S2, which can be confirmed by the 1 H NMR spectra (Supplementary Fig. S2B). The peaks at around chemical shifts (δ) of 5.3 and 5.6 ppm could be assigned to the acrylic protons (2 H) of the grafted methacryloyl group, and another peak at δ = 1.9 ppm could be attributed to the methyl group (3 H) of the grafted methacryloyl group. Meanwhile, there was a decrease of the intensity at 2.9 ≤ δ ≤ 3.1 ppm, which was assigned to the lysine methylene (2 H) marked as blue “b”. Taken together this confirms the successful synthesis of GelMA.

### Optimization of cell seeding in 3D scaffold

**Figure 1:**
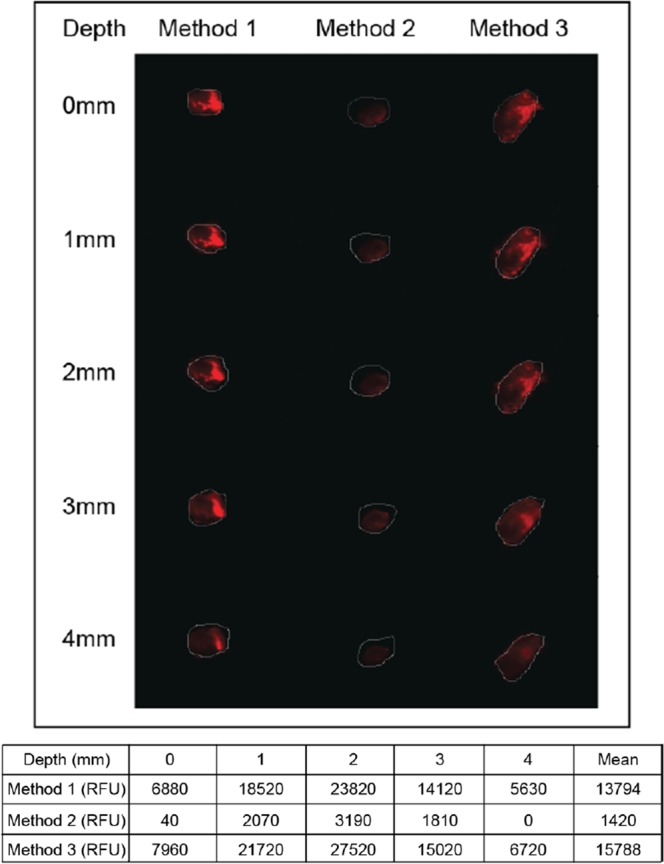
Study of cell culture growth conditions through infiltration into scaffolds. Depth imaging to examine cells (MKN74) penetration into 3D scaffold by indicated three methods as mentioned in the subheading of ‘Cells seeding and hybrid scaffold development’ under Methods section at depth of 0–4 mm. RFU of scaffold penetration into Method 1, MKN74 cells were pipetted onto scaffolds and immediately gelatinized; Method 2, scaffold were soaked in MKN74 cells for 10 min and gelatinized; Method 3, scaffold soaked in MKN74 cells for 10 min, transferred onto 24-well plate, incubated for 30 min and gelatinized. Finally, method 3 was selected based on the highest fluorescence intensity for subsequent studies.

To optimize cell seeding and infiltration, depth imaging of hybrid scaffold seeded was attempted using 3 different methods as showed in Fig. 1. MKN74 cells were detected at all depth when seeded using methods 1 and 3, as shown by higher relative fluorescent unit (RFU) when compared with method 2. Method 3 has the highest mean RFU (Fig. 1) indicating that more cells have penetrated the PLGA hybrid scaffold, after an incubation time of 30 min. It was conceivable that the hydrophobicity of the material prevented the efficient uptake of the cell suspension within the short duration of 10 min using method 2. Therefore, method 3 was used for subsequent experiments.

**Figure 2:**
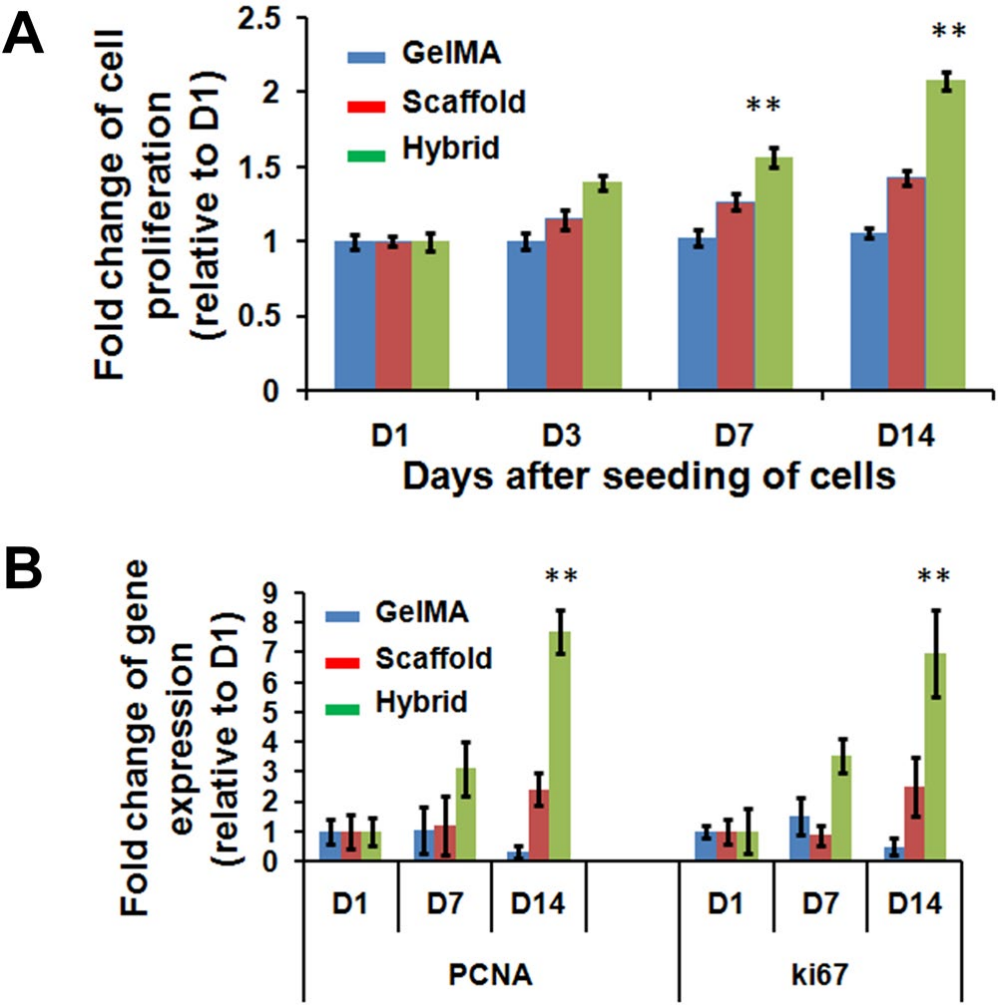
Proliferation rates of MDA-MB-231 cells in GelMA, scaffold and hybrid scaffold. **(A)** Fold changes of cell proliferation and **(B)** Gene expression of proliferation markers, PCNA and Ki67, were assessed at indicated (Day 1, 3, 7 and 14) time points after seeding the cells. β-actin was utilized to normalize gene expression data. Results were shown as mean ± S.D.

To evaluate the effects of seeding method on cell viability, we performed cell proliferation study using our hybrid 3D scaffolds (Fig. 2A). Our observation revealed that 3D hybrid scaffold significantly increased cellular proliferation at day 14 (D14) by >2-folds when compared with cells cultured in either PLGA scaffold or GelMA alone (Fig. 2A). This observation was further confirmed by a 6-folds higher expression level of proliferation markers, such as PCNA and Ki67 (Fig. 2B).

### Three-dimensional microenvironment induces EMT related genes expression

Nano-/microscale scaffolds surface topographies influence cell behaviors^32–35^ such as cell adhesion, migration, and proliferation; processes observed during cancer metastasis^36^. We hypothesized that the hybrid scaffold fabricated from PLGA and photopolymerizable hydrogel biomimetic features could provide a conducive 3D environment to promote EMT in the absence of soluble EMT-associated stimuli. To evaluate how dormant and aggressive cancer cells respond to this 3D system, we selected two cell lines with their different degree of the mesenchymal state. MKN74 (low-invasive) is a polarized gastric carcinoma and exhibited robust epithelial phenotype whereas MDA-MB-231 (highly invasive, basal-like) is a very aggressive mesenchymal type associated with poor prognosis.

**Figure 3:**
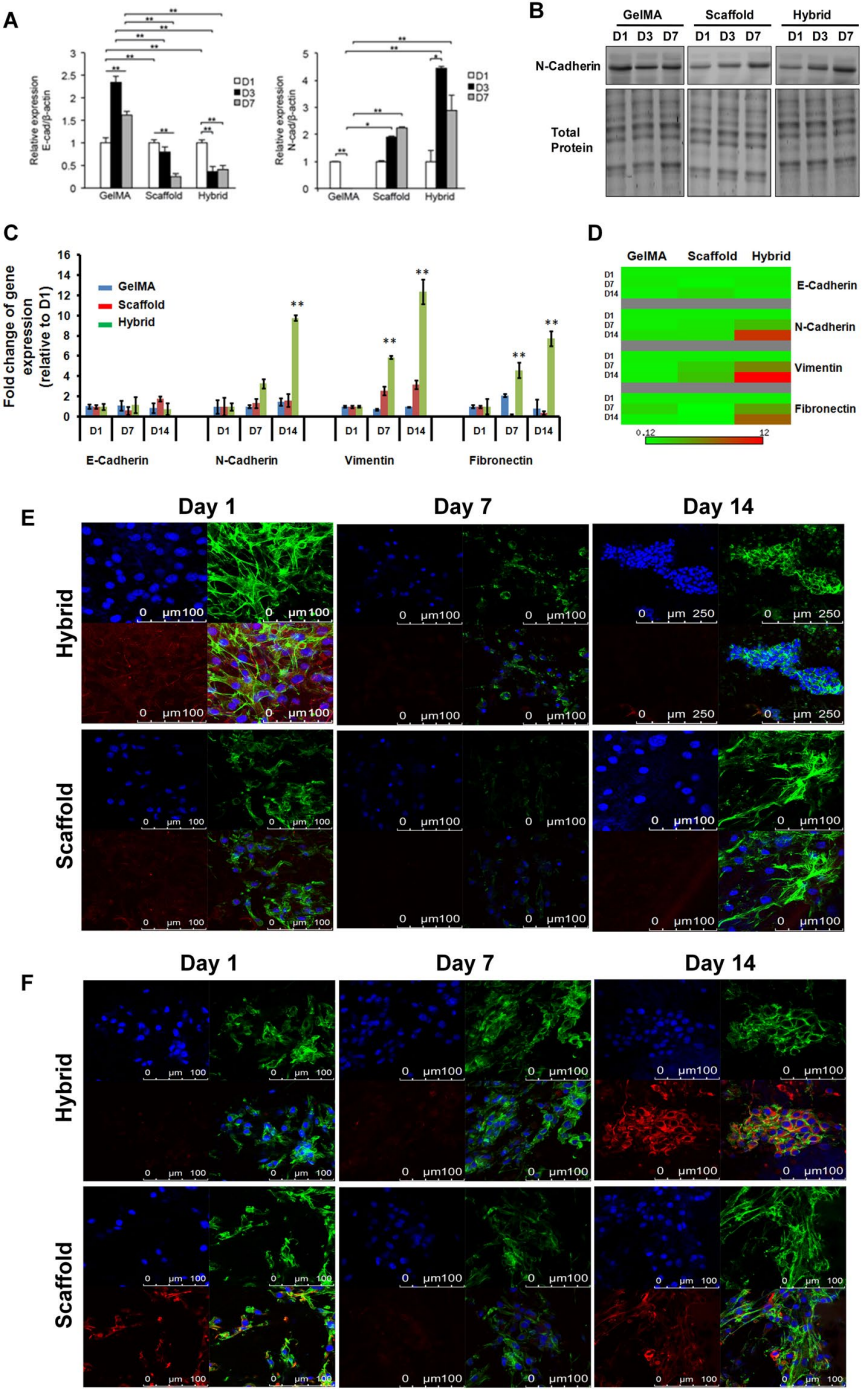
Relative transcript and protein expression levels of epithelial and mesenchymal markers using MDA-MB-231 and MKN74 cells in GelMA, scaffold and hybrid scaffold. **(A)** Transcriptional levels of EMT markers E-Cadherin and N-Cadherin were detected by qRT-PCR at Day1, Day3 and Day7 post culturing MKN74 cells. **(B**) Protein expression level of N-Cadherin of MKN74 cells at indicated time points, showing the up-regulation of N-Cadherin at D7 in hybrid scaffolds. We performed 3-independent experiments. One representative image has been shown here. Uncropped full images for total protein and western blot gels have shown in Supplementary Fig. S8. **(C**,**D)** Transcript levels of EMT markers E-Cadherin, N-Cadherin, Vimentin and Fibronectin in MDA-MB-231 cells at Day1, Day7 and Day14. β-actin was utilized to normalize gene expression data. Results were shown as mean ± S.D. **(E)** Representative confocal microscopic images of MDA-MB-231 cells stained with an epithelial marker, E-Cadherin (red) and Alexa Fluor 488 phalloidin for actin cytoskeleton (green) in scaffold and hybrid scaffold. Dual immunostaining of 3D hybrid scaffold cultured with cells showing aggregations of cells with minimal expression of E-Cadherin. **(F)** Representative confocal microscopic images of MDA-MB-231 cells stained with a mesenchymal marker, vimentin (red) and Alexa Fluor 488 phalloidin for actin cytoskeleton (green) in scaffold and hybrid scaffold. Dual immunostaining of 3D hybrid scaffolds showing higher expression level of vimentin.

Next, we compared the expression levels of EMT markers in MKN74 and MDA-MB-231 cells cultured for 1, 3 and 7 days in the hybrid scaffold, PLGA scaffold and GelMA alone. In contrast to GelMA, we detected a down-regulation of E-Cadherin expression in MKN74 cells cultured in PLGA scaffold and hybrid scaffold (Fig. 3A). The expression of N-Cadherin, a mesenchymal marker, was down-regulated in MKN74 cells embedded in GelMA matrix while its expression was up-regulated in the PLGA scaffold alone and hybrid scaffold (Fig. 3A). A higher protein expression level of N-Cadherin was also detected in cells cultured in the 3D hybrid scaffold (Fig. 3B). Similar observations were also obtained using MDA-MB-231 cells. Our results revealed a down-regulation of E-Cadherin and a significant up-regulation of mesenchymal markers vimentin, N-Cadherin, and fibronectin in MDA-MB-231 cells grown in 3D hybrid scaffold at D14 compared to PLGA scaffold and GelMA alone (Fig. 3C,D). Fluorescence imaging revealed lower expression level of E-Cadherin (Fig. 3E) and distinct aggregation of MDA-MB-231 cells in the hybrid scaffold, but not in PLGA scaffold alone. Thus, our hybrid scaffold promoted multi-cellular aggregation of fast-growing cells by clustering and facilitating their interactions. We observed that the mesenchymal marker vimentin expression level was higher in the hybrid scaffold at D14 compared to D1 (Fig. 3F). Consistently, our immunofluorescence images also showed higher expression level of another mesenchymal marker; N-Cadherin in hybrid scaffold using MDA-MB-231 cells at D14 as shown in Fig. S3. The EMT related markers, such as E-Cadherin, vimentin, N-Cadherin expressions in GelMA were not significantly changed in MDA-MB-231 cells (Fig. S4), suggesting that the transformation of epithelial to mesenchymal cells occurred due to both topographical and biochemical features of the hybrid scaffold. This was further supported by the lack of EMT related markers expression in the controls, i.e., fibrous scaffold as well as GelMA alone respectively where either the biochemical or topographical influence is missing.

**Figure 4:**
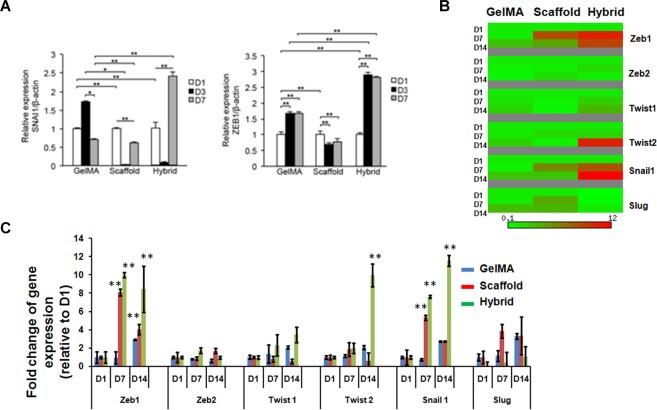
EMT regulatory transcriptional factors expression in MDA-MB-231 and MKN74 cells using GelMA, scaffold and hybrid scaffold. **(A)** Transcript levels of EMT regulatory transcriptional factors Snail1 and Zeb1 in MKN74 cells were detected by qRT-PCR at indicated time points. **(B,C)** Relative transcript expression levels of Zeb1, Zeb2, Twist1, Twist2, Snail1 and Slug in MDA-MB-231 cells. β-actin mRNA was utilized to normalize gene expression data. Results were shown as mean ± S.D.

Transcriptional reprogramming by key regulatory factors plays a critical role in EMT. Thus, we further evaluated the potential roles of 3D hybrid scaffolds in promoting expression changes of EMT regulatory markers in, firstly, MKN74 cells. We found that MKN74 cells cultured in hybrid scaffolds had a significantly higher expression of EMT regulatory markers SNAI1 and ZEB1 compared to PLGA scaffold and GelMA alone (Fig. 4A). Similarly, the transcript expression levels of SNAI1, ZEB1, and Twist2 were also elevated in MDA-MB-231 cells cultured in hybrid scaffold compared to scaffold and GelMA matrices alone (Fig. 4B,C). Immunofluorescence imaging confirmed the higher expression levels of EMT regulatory gene, SNAIL1 in the hybrid scaffold (Fig. S5), whereas the expression of SNAI1 was reduced in GelMA (Fig. S4). Overall, these results suggested that the 3D fibrous microenvironment with biochemical cues in hybrid scaffold might stimulate EMT in gastric and breast cancer cells as shown by the expression of EMT markers as well as the key regulatory factors.

### Three-dimensional microenvironment induces CSC-associated genes expression

**Figure 5:**
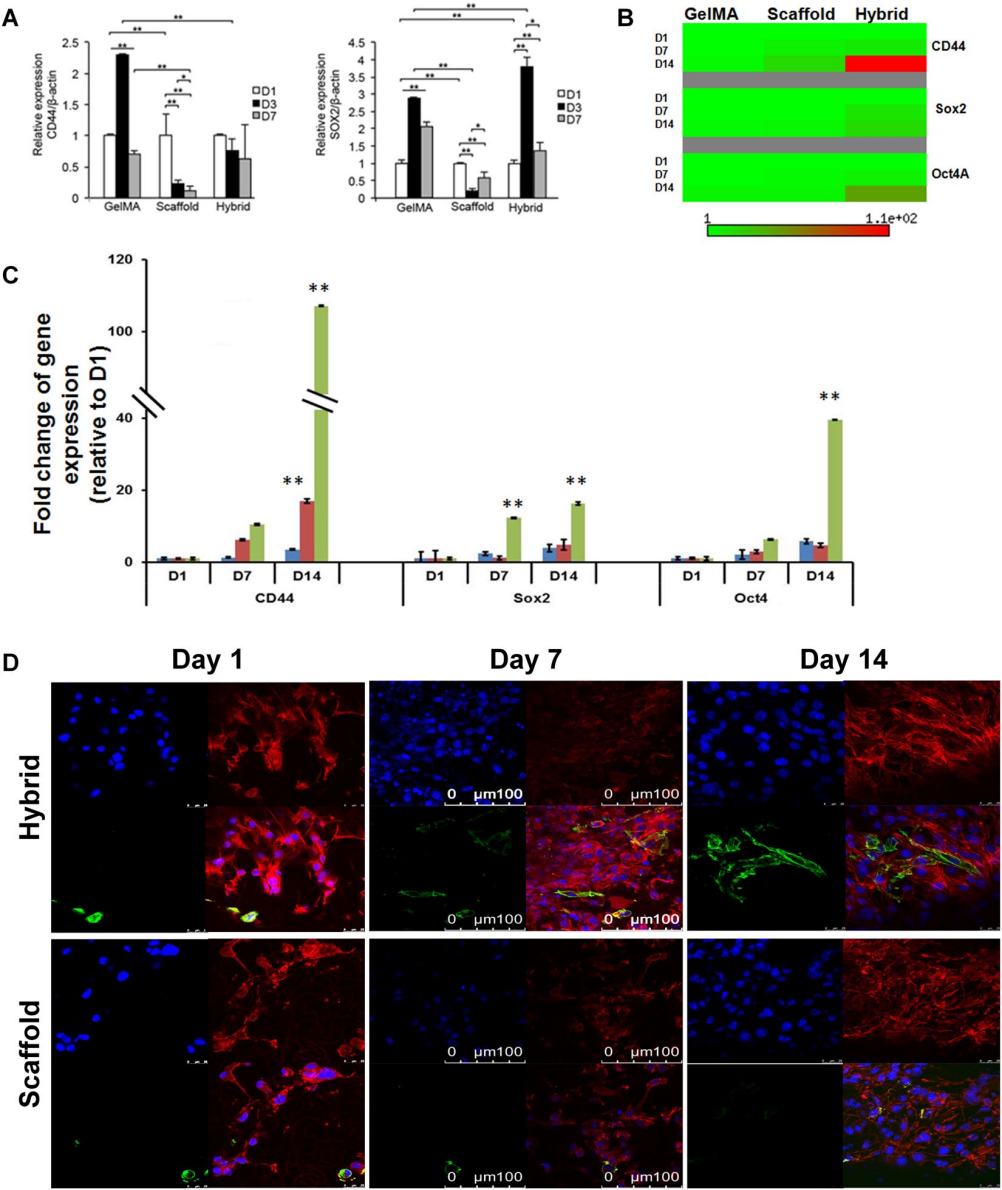
Relative transcript expression levels of cancer stem cells markers in MDA-MB-231 and MKN74 cells in GelMA, scaffold and hybrid scaffold. (**A)** Transcript expression levels of CSC markers CD44 and Sox2 in MKN74 cultured cells were measured by qRT-PCR at Day1, Day3, and Day7. **(B,C)** Transcript expression levels of CSC markers CD44, Sox2 and Oct4 in MDA-MB-231 cells at indicated time points. β-actin was utilized to normalize gene expression data. Results were shown as mean ± S.D. **(D)** Representative confocal microscopic images of cancer stem cells marker, CD44 (green) and Rhodamine-phalloidin for actin cytoskeleton (red) immunofluorescence using MDA-MB-231 cells in scaffold and hybrid scaffold. Dual immunostaining of 3D hybrid scaffolds showing higher expression level of CD44.

Cancer cells undergoing EMT exhibit stem cell-like characteristics^2^ and express biomarkers such as CD44, Sox2, and Oct4. Given that cancer cells undergoing EMT exhibit cancer stem cell-like phenotype, we were interested in examining the impact of our hybrid scaffold on the expression of stem cell markers. Therefore, MKN74 cells were cultured in our hybrid scaffold for 7 days to assess if the enhancement of CSC biomarkers, CD44 and Sox4, was due to their culture in a 3D environment. It was observed that there is an increase in the mRNA transcript levels of CD44 and Sox2 in GelMA matrix and down-regulation of those CSCs biomarkers in fibrous scaffold alone (Fig. 5A). We also observed that the expression of Sox2 was significantly up-regulated in the hybrid scaffold (Fig. 5A). We further verified the transcript expression levels of those CSCs markers using MDA-MB-231 cells. We found that the proportion of CD44+ MDA-MB-231 cells was ~12-folds higher in 3D hybrid scaffold compared to scaffold and GelMA alone, indicating that 3D hybrid scaffold strikingly influenced the phenotypic changes of breast cancer cells (Figs 5B–D, S4). Our immunofluorescence images showed that the expression levels of Sox2 and Oct4 in 3D hybrid scaffold were also higher than the scaffold and GelMA alone as shown in Figs S4, S6, S7. Taken together, our observations demonstrate that 3D hybrid scaffold promotes the conversion of non-CSCs to CSCs-like cells and may, therefore, have a potential role in enriching CSCs-like phenotypes.

## Discussion

Our study showed that a 3D hybrid scaffold potentiates EMT of a subpopulation of seeded cancer cells and supports the growth of other subpopulations of cancer cells. This heterogeneity highlights the strength of our 3D scaffold to realistically mimic the *in-vivo* situation, where a small population of cancer cells in a tumor would acquire the advantageous characteristics to undergo EMT, while the rest remain as the bulk of the primary tumor.

Various 3D tissue culture models using hydrogels based scaffolds^37,38^ have been developed to understand how 3D environment modify the behavior of cancer cells, as well as how cancer cells biomechanically interact with and respond to the 3D architecture. However, current 3D culture models to study EMT were mostly focused on gel systems that failed to fulfill the repertoire of ECM complexity such as topography and fibrous nature. Electrospun scaffolds which could simulate the physical topography of ECM have been developed for cancer studies and other tissue engineering studies. Several studies have reported the use of these scaffolds to culture tumoroids^39,40^. In the study by Girard *et al*., a PLGA based composite material was used to form the electrospun mats^39^. Mouse LLC1 (Lewis lung carcinoma) cells were cultured on the surface of the mat and cells eventually formed tight irregular aggregation of cells (tumoroids)^40^. In these studies, the tumoroids formed were “sitting on” the surface of the mats^39,40^. While tumoroids are evidently formed, most if not all, of the interactions are confined to 2D settings where only the base of tumoroids was interacting with the substrate. To the best of our knowledge, there is no study on tumoroids that were embedded and interacting in a true 3D manner with their ECM fibers. As shown by our results, such an environment together with the tuning of the biochemical properties is crucial for the induction of EMT-associated plasticity for different types of breast and gastric cancer cells.

EMT is the pivotal event to initiate cancer metastasis where epithelial cells adopt a migratory mesenchymal phenotype for invasion and distal dissemination by losing apical-basal polarity^1,2^. Thus far, the link between the EMT program and the CSC state has been derived largely from studies performed on 2D surfaces with their limited ability to reflect cell-ECM interactions. However, the development of novel *in-vitro* 3D cell culture model for the study of tumour progression and metastasis through EMT and enhancement of CSCs presents considerable challenges though essential for applications such as drug screening. In 3D microenvironment of our designed scaffolds, epithelial plasticity plays a key role in losing their epithelial junctions (as observed downregulation of E-cadherin expression) and switch to producing vimentin filaments (a mesenchymal marker). This EMT related functional hallmark allows the cells to gain more migratory and invading ability from stationary epithelial cells in the porous scaffold. We showed that our novel 3D hybrid scaffold of PLGA fibers with GelMA hydrogel was able to induce the occurrence of EMT and enhancement of CSCs in breast and gastric cancer cells as evidenced by the expression of related markers. Thus, our 3D hybrid scaffold comprised of PLGA fibers and cancer cells embedded in GelMA matrix acts as an abiotic/physical EMT inducer, leading to increased invasiveness and metastasis of cancer cells.

CSC subpopulations are particularly well poised to complete the metastatic cascade, thus CSCs are key targets for effective anticancer treatment^41^. It has been proposed that there are two alternative means of generating CSCs^42^. Intrinsic CSCs are thought to exist in primary tumors from the very early stages of tumorigenesis and maybe the oncogenic derivatives of normal-tissue stem or progenitor cells. Cancer cells undergoing EMT also exhibit CSCs-like phenotype, suggesting that among the heterogenic cancer cell population CSCs have a selective growth advantage in the 3D microenvironment. A subpopulation of cancer cells embedded in our 3D scaffold acquired CSC-like markers, which conceivably represent cancer cells during EMT. EMT is accompanied by down-regulation of epithelial marker, E-Cadherin, and up-regulation of mesenchymal markers, N-Cadherin, vimentin, and fibronectin^43,44^. The regulatory factors, SNAI1 and ZEB1, are repressors of E-Cadherin^45^. We observed changes in gene expression reminiscent of cancer cells undergoing EMT in 2D when induced by EMT stimuli. Remarkably, in our 3D hybrid scaffold, the EMT was triggered in the absence of any classical stimuli such as TGF-b1 and hypoxia^46^. However, similar efficacy was not achieved when cells were cultured in PLGA scaffold or GelMA alone. The same 3D hybrid scaffold also supported the growth of other subpopulations cancer cells. This heterogeneity further highlights the strength of our 3D scaffold to realistically mimic the *in-vivo* situation, where tumors are heterogeneous populations of cells. Regardless of xenograft or patient tumors, only a small population of cancer cells will acquire the advantageous characteristics to undergo EMT, while the rest remain as the bulk of the primary tumor.

Clearly, the appropriate combination of fibrous architecture and protein matrix is important to influence the behaviour of cancer cells^47,48^. The relatively hydrophobic nature of the PLGA polymers^31^ makes it challenging for cells to penetrate through the pores, which was evident in the experiment using the fibrous scaffold alone. The incorporation of GelMA overcomes this limitation. GelMA is modified based on gelatin, a denatured collagen, functionalized with methacrylamide side groups that facilitate cross-linking upon a photo-initiated reaction by ultraviolet (UV)^49,50^. Gelatin retains the natural integrin-binding motifs such as arginine-glycine-aspartic acid (RGD)-peptide that have a profound effect on the ability of cells attachment onto the fibers, and matrix metalloproteinases (MMP) degradable sites, enabling cell-mediated ECM remodeling and migration within the 3D microenvironment^51^. Moreover, the presence of methacrylamide side group allows the refinement of density and stiffness of the hydrogel by tweaking the UV intensity and photo-initiation concentration^51^.

It is well established that the physical and chemical properties of surrounding ECM in normal tissue is different from tumor. For one, increased tissue stiffness is a classic characteristic of solid tumors. Cancer cells biomechanically interest with and respond to the stiffness of the ECM. Therefore, reengineering of the physical microenvironment of tumor to improve drug delivery and efficacy has been gathering interest. As 90% of cancer-related death is attributed to metastasis, our work herein contributes to this global research direction, on physical microenvironment on EMT of cancer cells. Our developed 3D hybrid scaffold allows studying the effect of abiotic/physical component of tumor microenvironment in promoting aggressiveness of the cancer, without the confounding influence from other EMT inducer like TGF-β. This hybrid platform provides a realistic and straightforward model to investigate the role of the physical and biochemical microenvironment of scaffold on the enrichment of CSCs *in-vitro* and anti-cancer drug screening. The platform also allows scientists to study mechanosignaling in tumor progression in 3D.

## Conclusions

In this study, we have designed and fabricated a 3D hybrid scaffold comprised of PLGA fibers and GelMA hydrogel by using electrospinning and photopolymerization techniques to study EMT in breast and gastric carcinoma cells. This hybrid scaffold provided new dimensions of 3D environment where the tumoroids or cells could interact with the environment in all surfaces of the cells/tumoroids. Quantitative transcript and translational analysis revealed that the tumoroids in 3D hybrid scaffold exhibited significant up-regulation of EMT markers compared to those in the scaffold and GelMA alone, indicating that the hybrid scaffold provided strong topographical and biochemical influence on tumour progression and metastasis. In addition, our findings also demonstrate the enhancement of CSCs population in a 3D *in-vitro* cell culture model. Overall, we anticipate that this 3D hybrid scaffold could be a useful and more realistic model for understanding CSC biology and anti-cancer therapy.

## Methods

### Reagents and antibodies

High glucose Dulbecco’s Modified Eagle Medium (DMEM) and RPMI-1640 were purchased from Lonza (Basel, Switzerland). Fetal bovine serum (FBS) and antibiotic-antimycotic were purchased from Gibco (Fisher Scientific, Waltham, MA, USA). E-Cadherin, N-Cadherin, vitronectin, fibronectin, SNAI1 antibodies were from Cell Signaling Technology, USA; Alexa-fluor Goat anti-rabbit 594, Alexa-fluor Goat anti-mouse 488, Alexa Fluor 488 Phalloidin and Rhodamine phalloidin were from Thermo Fisher Scientific, USA. Fluoroshield mounting medium with DAPI was purchased from Abcam (Cambridge, UK). Unless otherwise indicated, all reagents were obtained from Sigma-Aldrich.

### PLGA fibrous scaffold preparation

Poly Lactic-co-Glycolic Acid (PLGA) was dissolved in a solvent mixture ratio of chloroform (CF): dimethylformamide (DMF) = 7:3 (v/v). The polymer solution was then transferred into a 5 mL syringe connected to a 21 G blunt needle (BD precision guide) for electrospinning. The polymer was dispensed using a syringe pump at a constant flow rate of 0.5 mL/h in a humidity controlled chamber. A stationary grounded F108 containing buffer solution (ethanol:water = 7:3, v/v, 0.05% F108) in the collector was placed at a distance of 3–4 cm from the tip of the needle for obtaining random fibers. Electrospinning of the polymer was carried out by applying a positive voltage of 18 kV between the needle tip and the stationary collector. The electrospun PLGA scaffold was kept overnight on a freeze dryer in order to remove residual solvents. PLGA scaffolds were sterilized by immersing in 70% ethanol for 20 min followed by UV irradiation at 254 nm for 1 h for subsequent studies.

### GelMA synthesis

GelMA was prepared in accordance with our previous papers^52,53^. Briefly, Type A gelatin (175 bloom, Sigma Aldrich) was dissolved at 60 °C in phosphate buffered saline (PBS, Sigma-Aldrich), at pH 8.0. GelMA was prepared by reaction of free amino groups of gelatin lysine with methacrylic anhydride (MAA, Sigma Aldrich, 94%) at a 1:6.6 molar ratio of amine groups to MAA at 50 °C for 3 hours, followed by adjusting the pH 7.4 using 5 M sodium hydroxide. It was then filtered at 0.2 um, dialyzed using PALL Minimate TFF capsule with 10 kDa MWCO at 40 °C for 1 day, lyophilized, and finally stored at −20 °C until further use. The degree of substitution of GelMA was calculated from H-NMR results^49,52,53^.

### Cell culture and maintenance

Human breast carcinoma, MDA-MB-231 (American Type Culture Collection, Rockville, Maryland, USA) and gastric carcinoma, MKN74 (Japanese Collection of Research Bioresources Cell Bank, Osaka, Japan) were cultured in DMEM and RPMI-1640 cell culture medium, respectively. Both media were supplemented with 10% fetal bovine serum, 100 IU/mL penicillin (Sigma) and 0.1 mg/mL streptomycin (Sigma). Cells were grown at 37 °C in a humidified incubator with 5% CO_2_.

### Cells seeding and hybrid scaffold development

The hybrid scaffold was prepared by impregnating GelMA into the electrospun scaffolds. PLGA scaffolds were trimmed into 5 mm × 5 mm × 5 mm dimensions. GelMA solution was prepared by 8% w/v GelMA in PBS with 0.1% w/v Irqacure 2959. Gelation of GelMA solution was processed by UV irradiation at 365 nm for 10 min. To monitor cells infiltration, the cells (MKN74) were stained with SYTO® 60 red fluorescent nucleic acid stain (Thermo Fisher Scientific, USA) for 10 min and washed with PBS before seeding.

Three different methods for infiltration of cells were firstly attempted:

Method 1: 50 μL of MKN74 (1 × 10^7^ cells/mL) cells in GelMA solution was soaked into PLGA scaffold and immediately gelatinized using 365 nm UV for 10 min.

Method 2: 50 μL of MKN74 (1 × 10^7^ cells/mL) cells in GelMA were soaked into PLGA scaffold for 10 min followed by gelatinized using 365 nm UV for 10 min.

Method 3: PLGA scaffold was soaked with 50 μL of MKN74 (1 × 10^7^ cells/mL) cells in GelMA for 10 min and then transferred onto 24 wells plate followed by incubation for another 30 min and gelatinized.

PLGA scaffold and GelMA hydrogel alone were used as a control. For PLGA scaffold matrix: 50 μL cells suspension (1 × 10^7^ cells/mL) in culture medium was soaked into PLGA scaffold for 10 min and then transferred onto a 24 well plate and incubated for 30 min at 37 °C. For GelMA matrix: 50 μL of cells (1 × 10^7^ cells/mL) were suspended into GelMA solution and were photo crosslinked.

2-mL of media supplemented with 100 IU/mL penicillin, and100 μg/mL streptomycin were added into each well of 24 well plates for hybrid scaffold as well as its respective controls and incubated at 37 °C in a humidified incubator with 5% CO_2_. Finally, depth optical imaging of scaffolds was analysed using the Odyssey CLx Li-Cor scanner, USA. Based on the fluorescence intensity of seeded cells in the scaffold, method 3 has shown to support the best cell infiltration and therefore method 3 will be used for subsequent studies.

### Cell proliferation analysis

Cell proliferation was determined using the Alamar Blue assay following the manufacturer’s protocol (Thermo Fisher Scientific). Briefly, cells were washed with PBS before adding 1 mL of Alamar Blue solution (110 mg Resazurin per 1 mL medium) to each well. After 2 h incubation, the solution was transferred to a black-bottom 96-wells plate to measure the fluorescence intensity. The cell number was calculated based on standard curves created for cells grown as monolayers.

### Microscopy

For fluorescence microscopy, cells on scaffolds were rinsed with PBS and fixed with 4% paraformaldehyde for 10 min at room temperature. Actin cytoskeleton was stained with Alexa Fluor 488 phalloidin (green) or Rhodamine–phalloidin (red). Fixed cells in scaffolds were blocked with 5% BSA for 1 h, followed by incubation with primary antibody (1:100 in 5% BSA) overnight at 4 °C. Samples were washed thrice with PBS before incubation with either Alexa-fluor Goat anti-rabbit 594 or Alexa-fluor Goat anti-mouse 488 secondary antibodies (1:100 dilutions in 5% BSA) for 2 h at 4 °C. Samples were mounted using Prolong Gold anti-fade reagent containing 4′,6-diamidino-2-phenylindole (DAPI) as a counterstain to visualize cell nuclei. Images were captured using confocal microscope with the appropriate filters using a Nikon Ri1 Color Cooled Camera System and 40x Oil Objective Lens (Nikon Instruments, Melville, NY). For scanning electron microscopy (SEM) analysis, fibers of scaffolds were freeze-dried, sputter coated with gold for 30 s and imaged at 5 kV with JEOL 6360 SEM. The average diameter of the fibers was calculated by analyzing SEM images with ImageJ software (NIH, USA).

### RNA extraction and RT-qPCR

Total RNA was extracted from the samples according to the manufacturer’s instructions (Omega Bio-Tek, USA). Samples were washed with PBS and soaked into 350 μL lysis buffer and vortexed. The quality and quality of RNA were measured with NanoDrop Spectrophotometer (NanoDrop Technologies Inc., USA). A total of 1 ug RNA was used for cDNA synthesis using qScript™ cDNA SuperMix kit (Quanta Biosciences, USA). Quantitative real-time PCR was performed as previously described (Pal *et al*., 2011). In brief, qRT-PCR was performed using CFX96 real time PCR detection system (Bio-Rad Laboratories, Inc, USA). The reaction mix comprises of 10 μL of KAPA SYBR FAST master mix (2×) universal, 0.4 μL of each forward and reverse primers (10 μM), 1 μL of diluted cDNA and PCR grade water to make up a final volume of 20 μL. The following cycling thermal profile was employed: enzyme activation at 95 °C for 3 min, 35 cycles of 95 °C for 3 s and 60 °C for 20 s followed by a dissociation step to analysis the melt curve of the amplified product. ΔΔCt values of triplicates were normalized using endogenous control β-actin, and relative expression was calculated. List of primers used is stated in Supplementary Table S1.

### Protein extraction and immunoblot analysis

Total protein was extracted from the samples using M-Per® Mammalian Protein Extraction Reagent (Thermo Fisher, USA) at different indicated days. Samples were washed with PBS followed by protein extraction into 200 μL protein extraction reagent. Equal amounts of protein extracts were resolved by 12% SDS-PAGE and electrotransferred onto polyvinylidene difluoride membranes. Membranes were processed according to standard procedure^54,55^ and proteins were detected by Odyssey CLx Li-Cor scanner. A Coomassie blue-stained membrane was used to check for equal loading and transfer.

### Statistical analysis

Statistical analyses were performed using two-tailed Mann-Whitney tests with SPSS software. A p-value of ≤0.05 was considered significant.

## Supplementary information

Supporting Information
