# Supplementary material for: Epithelial-mesenchymal transition of cancer cells using bioengineered hybrid scaffold composed of hydrogel/3D-fibrous framework
Source: Sci Rep. 2019 Jun 20;9:8997. doi: 10.1038/s41598-019-45384-9 (PMC6586872; doi:10.1038/s41598-019-45384-9)

Epithelial-mesenchymal transition of cancer cells using bioengineered hybrid scaffold composed of hydrogel/3D-fibrous framework

Mintu PAL[1,2,#], Huizhi CHEN[1,#], Bae Hoon LEE[1,3], Justin Yin Hao LEE[4], Yun Sheng YIP[5], Nguan Soon TAN[4,5*], Lay Poh TAN[1*]

Supporting information

Supplementary Table

Gene Name	Forward Primer (5´ -3´)	Reverse Primer (5´ -3´)
β-actin	GGAACGGTGAAGGTGACAG	TGGACTTGGGAGAGGACT
E-Cadherin	CCCACCACGTACAAGGGTC	CTGGGGTATTGGGGGCATC
PCNA	CCTGCTGGGATATTAGCTCCA	CAGCGGTAGGTGTCGAAGC
Ki67	GCCTGCTCGACCCTACAGA	GCTTGTCAACTGCGGTTGC
N-Cadherin	ACGCTCTCCCTCCCTGTT	TAATGAGGACCACGCTCAGG
Vimentin	CGCCAGATGCGTGAAATGG	ACCAGAGGGAGTGAATCCAGA
Fibronectin	CAGTGGGAGACCTCGAGAAG	TCCCTCGGAACATCAGAAAC
Twist1	GTCCGCAGTCTTACGAGGAG	GCTTGAGGGTCTGAATCTTGCT
Twist2	GCAAGAAGTCGAGCGAAGAT	GCTCTGCAGCTCCTCGAA
Snail1	TTCTCTAGGCCCTGGCTGC	TACTTCTTGACATCTGAGTGGG TCTG
Slug	CTGGGCTGGCCAAACATAAG	CCTTGTCACAGTATTTACAGCT GAAAG
Zeb1	TGTGAATGGGCGACCAAGA	GTGGGACTGCCTGGTGATG
Zeb2	GCCGAGTCCATGCGAACT	CCATGATCGGCTGCTTCAT
CD44	TCACATTAAGTTTGCATGACCTG	AAGTATCTCCGACCGGGATAA
SOX2	CCCCTTTATTTTCCGTAGTTGTA TTT	AACTTAGTCAGACGGCTCTTAG
Oct4	GTGTTCAGCCAAAAGACCATCT	GGCCTGCATGAGGGTTTCT

Table S1: List of Primer sequences used for qRT-PCR

Supplementary Figures.

Figure S1: Synthesis and characterization of electrospun fibrous scaffolds. **(A)** Representative SEM images of electrospun fibers with an average diameter of 1.6±0.13 um of 3D porous scaffolds. Distribution of fiber (**B**) diameter and (**C**) pore size within 3D electrospun fibrous scaffolds.

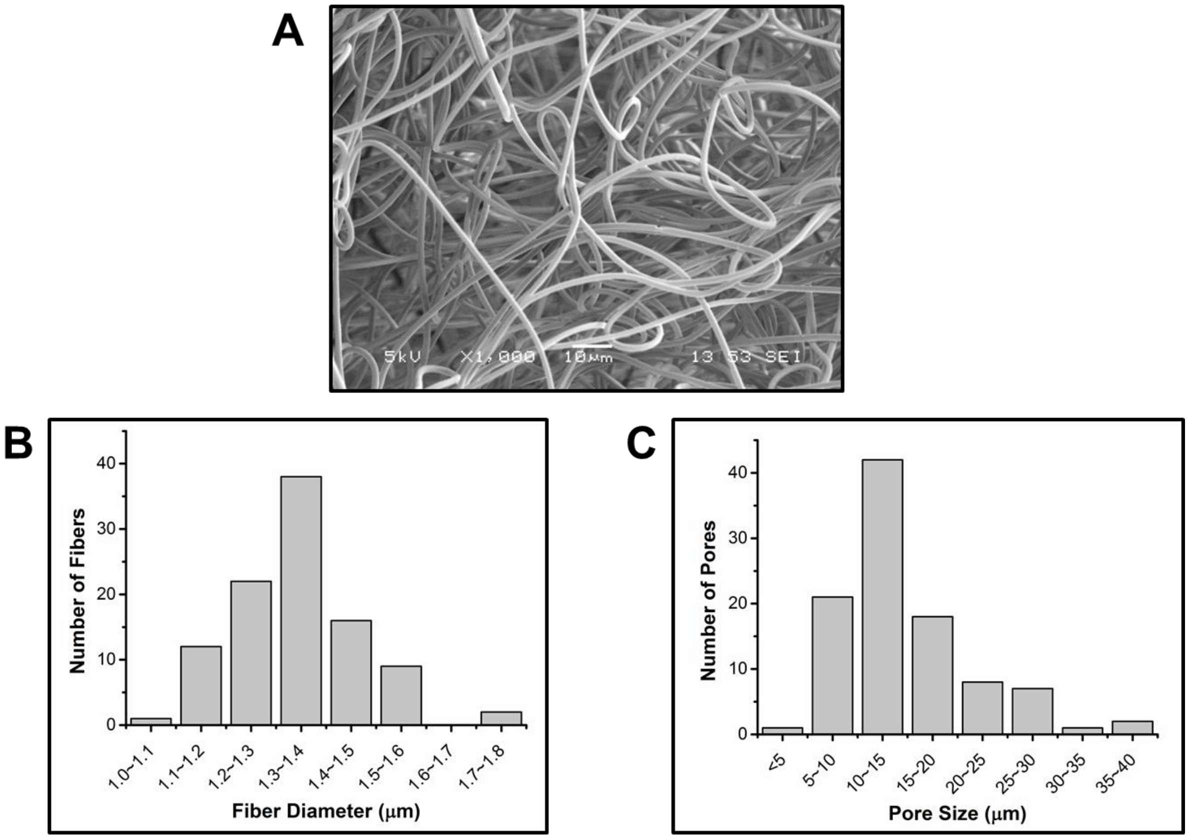

Figure S2: The chemical structures (A) of unmodified gelatin and Gelatin methacrylate (GelMA), and their respective 1H-NMR spectra (B). Orange “a” and green “c” represent the signals of the acrylic protons and methyl group of the grafted methacrylic group respectively, and blue “b” indicates the signal of lysine methylene.

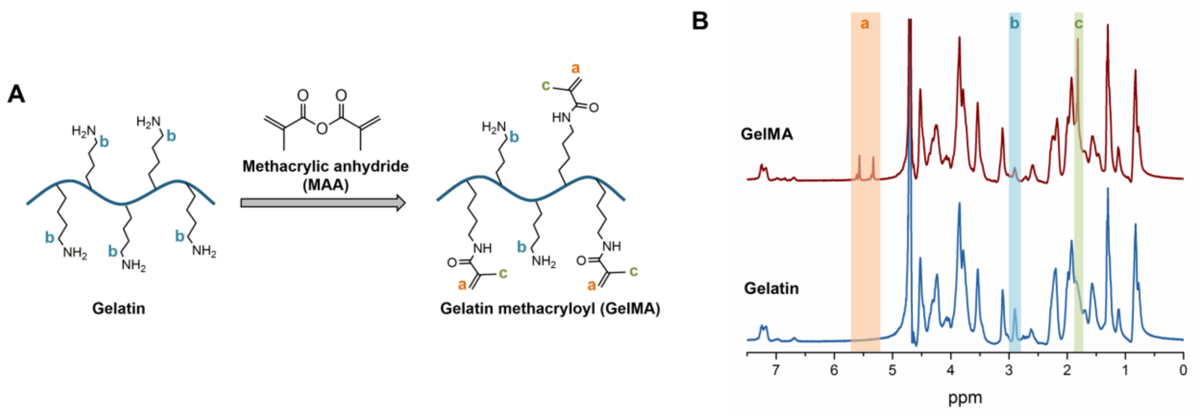

Figure S3: Representative confocal microscopic images of MDA-MB-231 cells stained with mesenchymal marker, N-Cadherin (red) and Alexa Fluor 488 phalloidin for actin cytoskeleton (green) in 3D hybrid scaffold.

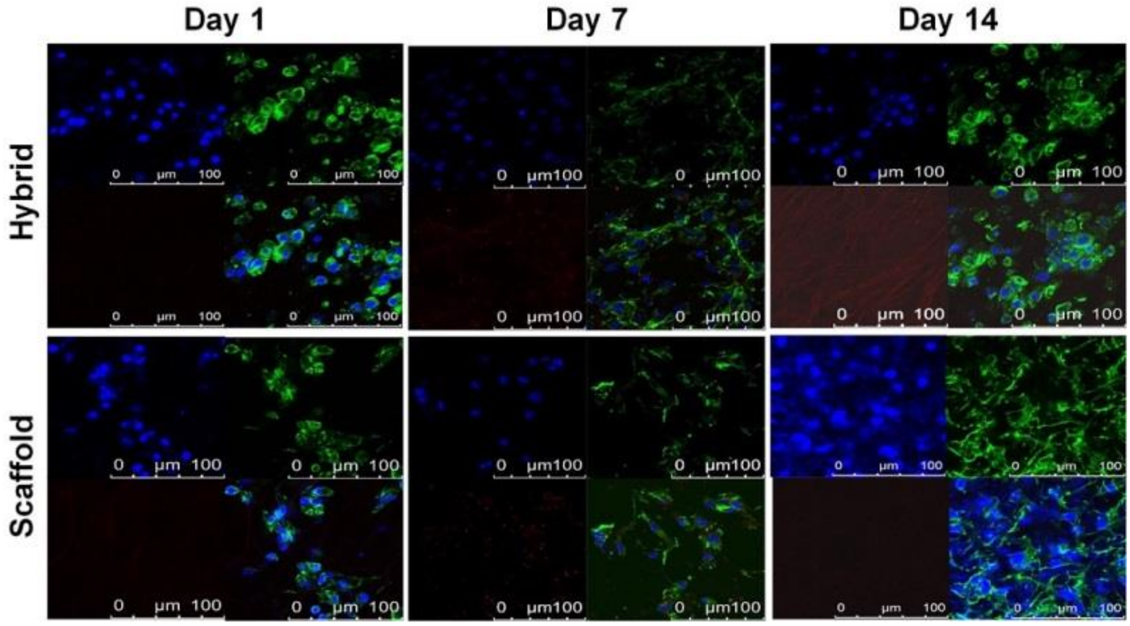

Figure S4: Representative confocal microscopic images of MDA-MB-231 cells stained with E-Cadherin, vimentin, N-Cadherin, CD44, Sox2, Oct4 and Snail1 in GelMA.

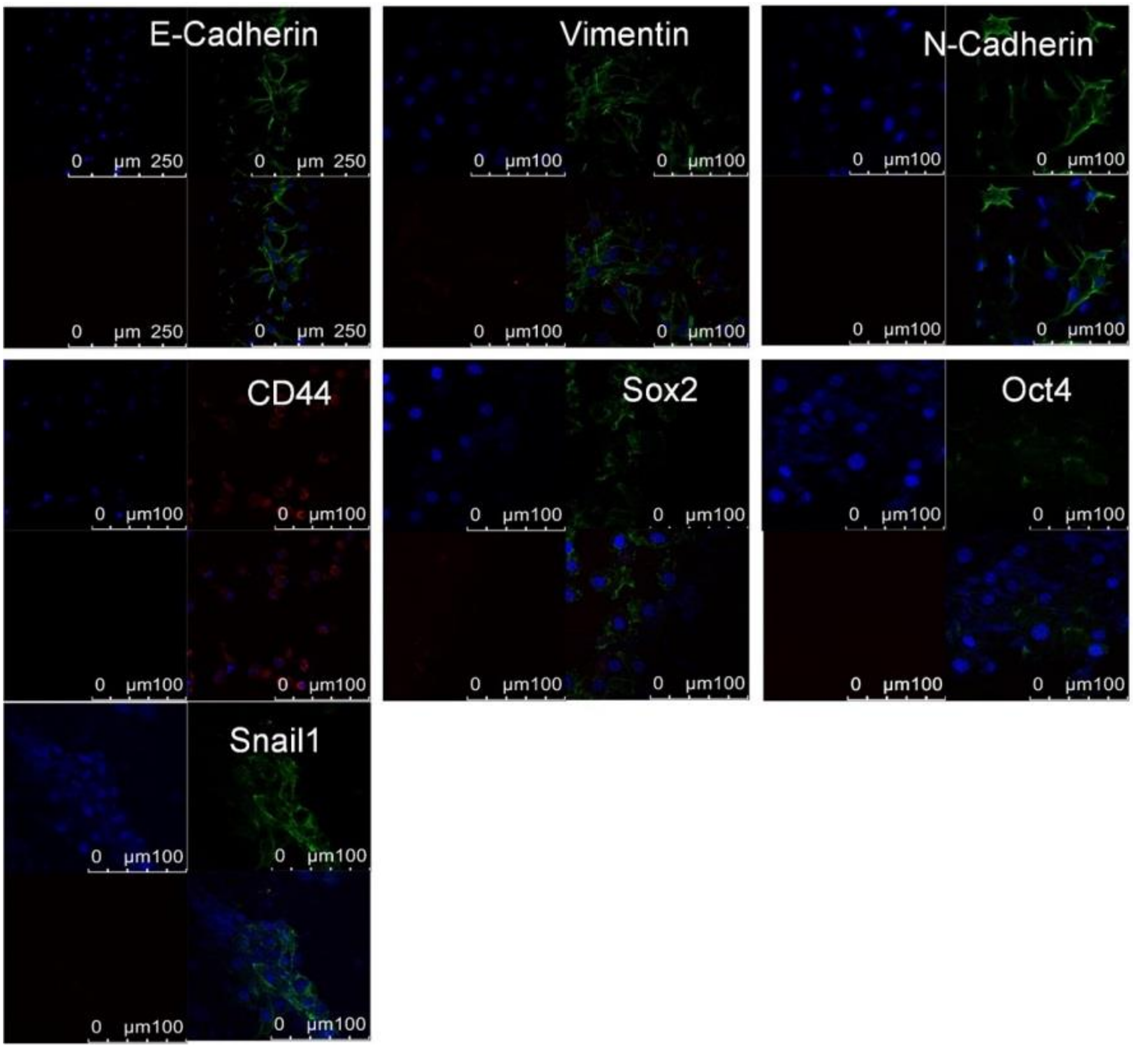

Figure S5: Representative confocal microscopic images of MDA-MB-231 cells stained with transcription regulatory marker, Snail1 (red) and Alexa Fluor 488 phalloidin for actin cytoskeleton (green) in 3D hybrid scaffold. Dual immunostaining of 3D hybrid scaffolds cultured with cells showing higher expression level of Snail1.

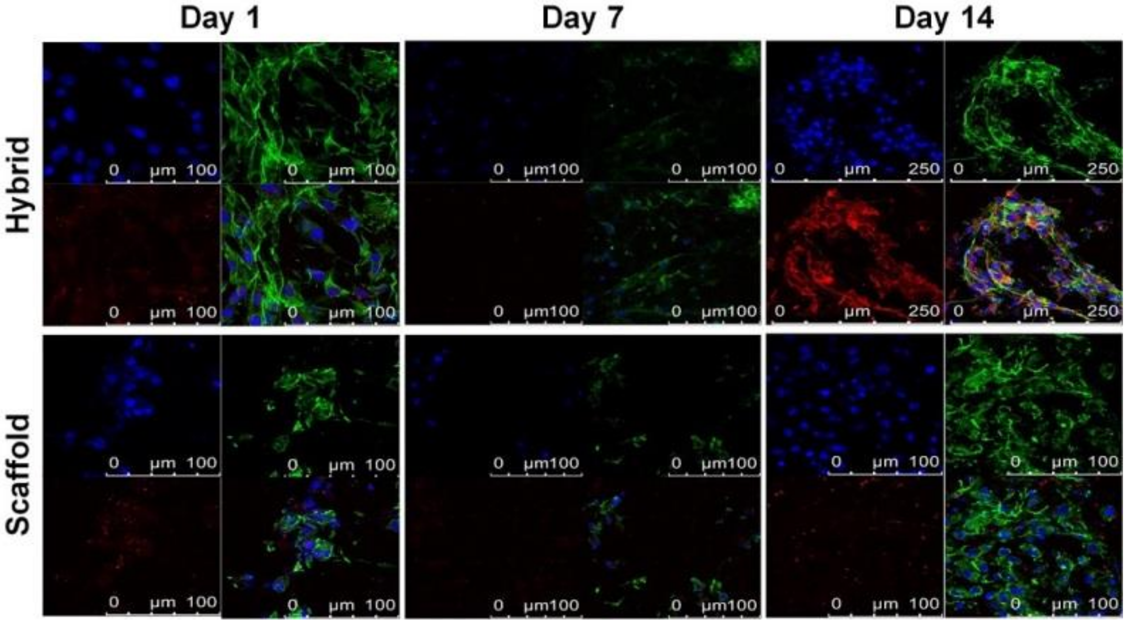

Figure S6: Representative confocal microscopic images of MDA-MB-231 cells stained with cancer stem cells marker, Sox2 (red) and Alexa Fluor 488 phalloidin for actin cytoskeleton (green) in 3D hybrid scaffold. Dual immunostaining of 3D hybrid scaffolds showing higher expression level of Sox2.

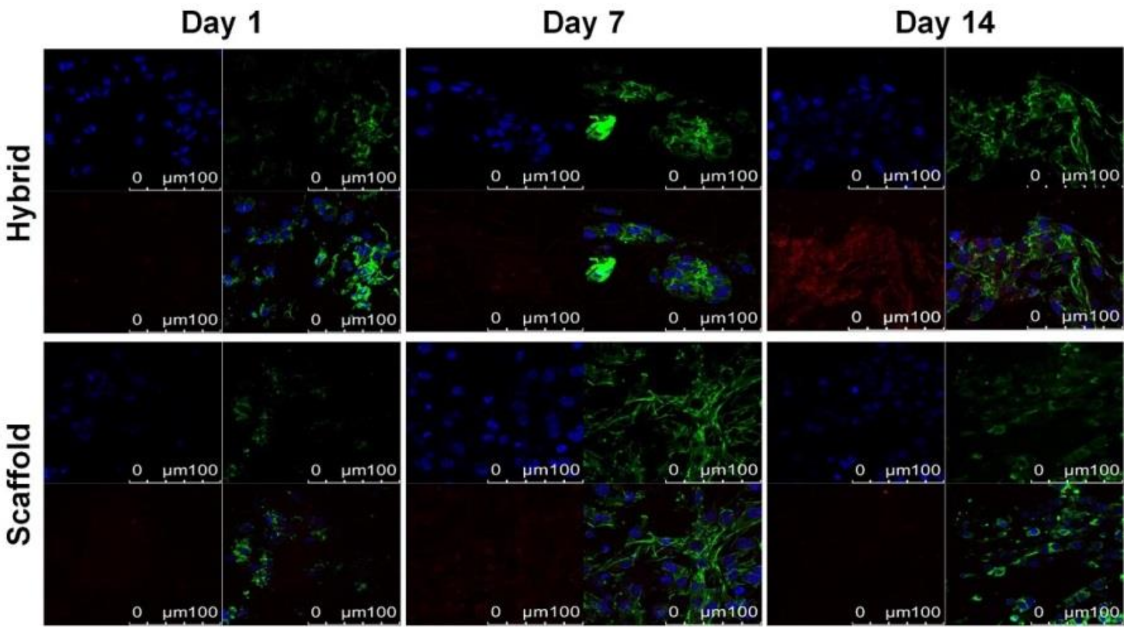

Figure S7: Representative confocal microscopic images of MDA-MB-231 cells stained with cancer stem cells marker, Oct4 (red) and Alexa Fluor 488 phalloidin for actin cytoskeleton (green) in 3D hybrid scaffold. Fluorescence immunostaining of Oct4 is showing higher expression level in hybrid scaffolds.

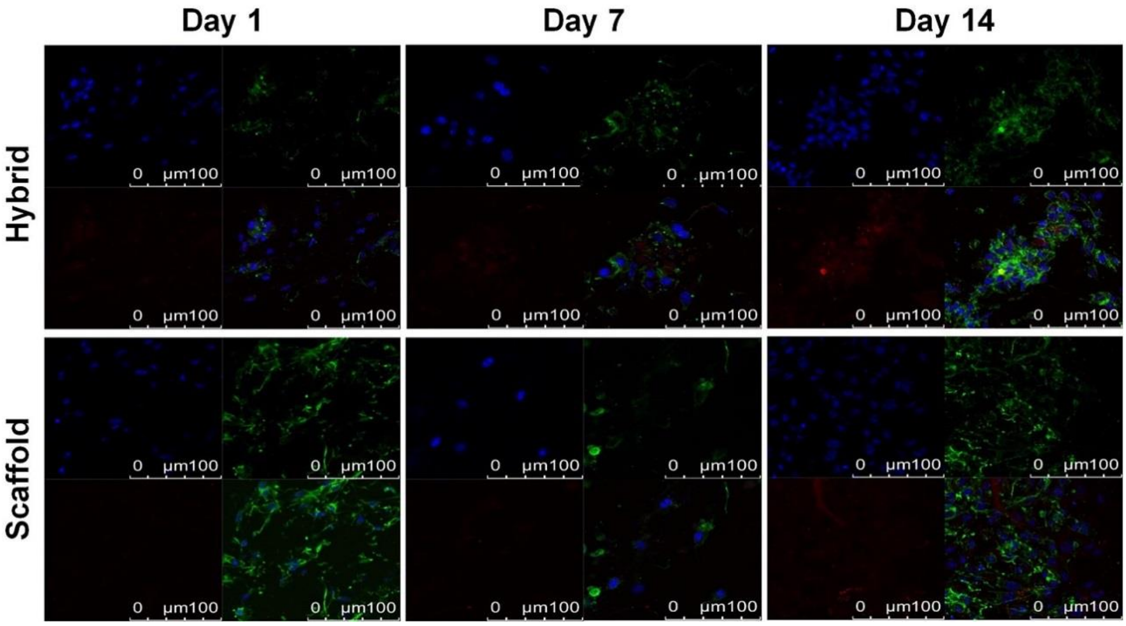

Figure S8. Uncropped full images for total protein and western blot gels. Broken boxes mark the borders of the final cropped images shown in Figure 3B.

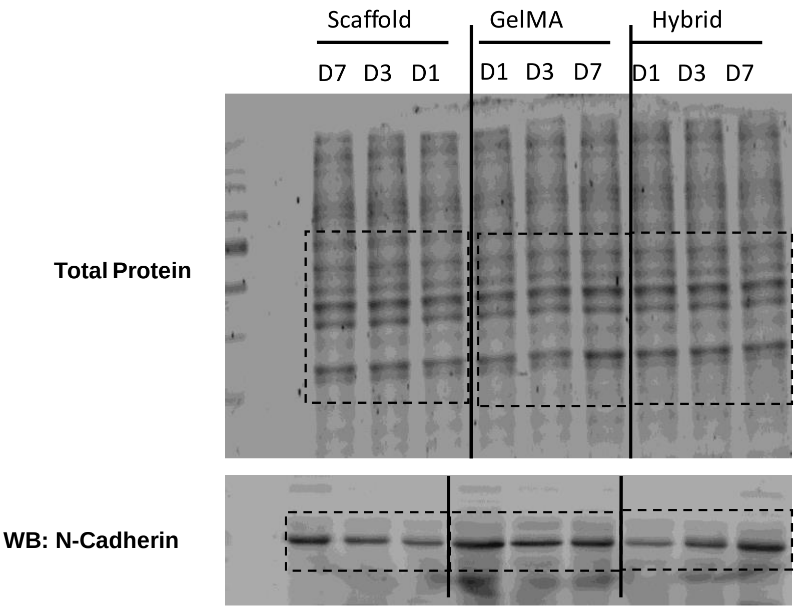

Supplement: Supplementary file 1 — Supporting Information [file 41598_2019_45384_MOESM1_ESM.pdf]
